# Complex Genomic Rearrangements Involving *ETV6*::*ABL1* Gene Fusion in an Individual with Myeloid Neoplasm

**DOI:** 10.3390/genes14101851

**Published:** 2023-09-23

**Authors:** Zhongxia Qi, Catherine Smith, Neil P. Shah, Jingwei Yu

**Affiliations:** 1 Department of Laboratory Medicine, University of California San Francisco, San Francisco, CA 94107, USA; 2 Department of Medicine, University of California San Francisco, San Francisco, CA 94143, USA

**Keywords:** *ETV6*::*ABL1* gene fusion, myeloid neoplasm, genomic rearrangement, additional anomalies, mate-pair sequencing

## Abstract

*ETV6*::*ABL1* gene fusion is a rare recurrent genomic rearrangement associated with hematologic malignancies, and frequently occurs with additional anomalies. Due to the opposite chromosome orientations of the *ETV6* and *ABL1* genes, an oncogenic in-frame *ETV6*::*ABL1* gene fusion cannot be formed by a simple translocation. The molecular mechanism of the *ETV6*::*ABL1* fusion and the significance of co-occurring anomalies are not fully understood. We characterized genomic alterations in an individual with *ETV6*::*ABL1* gene-fusion-positive myeloid neoplasm using various genomic technologies. Our findings uncovered a molecular mechanism of the *ETV6*::*ABL1* fusion, in which a paracentric inversion within the short arm of chromosome 12 (12p) and a translocation between the long arm of a chromosome 9 and the 12p with the inversion were involved. In addition, we detected multiple additional anomalies in the individual, and our findings suggested that the *ETV6*::*ABL1* fusion occurred as a secondary event in a subset of cells with the additional anomalies. We speculate that the additional anomalies may predispose to further pathogenic changes, including *ETV6*::*ABL1* fusion, leading to neoplastic transformation.

## 1. Introduction

*ETV6*::*ABL1* gene fusion is a rare but recurrent genomic rearrangement that has been reported in various hematologic neoplasms, including myeloproliferative neoplasms (MPNs), acute lymphoblastic leukemia (ALL), and acute myeloid leukemia (AML), frequently with eosinophilia [1,2,3,4,5,6]. An in-frame *ETV6*::*ABL1* gene fusion cannot be formed by a simple chromosome translocation due to the opposite orientations of *ETV6* and *ABL1* on their host chromosomes 12 and 9, respectively. As a result, at least three breaks are required to generate a functional in-frame *ETV6*::*ABL1* fusion gene. Additional anomalies in affected cells are frequent but show no consistent features. It has been known that the ETV6::ABL1 fusion activates the tyrosine kinase activity of ABL1, promotes neoplastic transformation similar to the BCR::ABL1 fusion, and confers sensitivity to ABL1 tyrosine kinase inhibitors (TKIs) [6,7,8,9]. However, detailed molecular characterizations of *ETV6*::*ABL1* fusion and co-occurring additional anomalies are limited, and the molecular mechanism of this heterogeneous group of abnormalities is not fully understood.

In this study, we characterized genomic alternations in an individual with *ETV6*::*ABL1*-positive myeloid neoplasm using chromosome banding, fluorescence in situ hybridization (FISH), and whole-genome mate-pair sequencing (MP-seq). Our findings uncovered a novel molecular mechanism of forming a functional in-frame *ETV6*::*ABL1* fusion gene. We also detected multiple additional co-occurring anomalies with the *ETV6*::*ABL1* gene fusion, and discussed the significance of these additional anomalies and potential hierarchy of alterations in this individual.

## 2. Materials and Methods

### 2.1. Chromosome Analysis

Bone marrow chromosome analysis was performed following the standard cytogenetic methods. Chromosome abnormalities were described according to the International Cytogenomic Nomenclature 2020 [10].

### 2.2. FISH

Interphase and metaphase FISH analyses were performed following a standard protocol (https://www.molecular.abbott/us/en/vysis-fish-knowledge-center, accessed on 10 January 2020). *BCR*/*ABL1* Dual Color Dual Fusion and *ETV6* Dual Color Break Apart probe kits were purchased from Abbott Molecular (Des Plaines, IL, USA). A bacteria artificial chromosome (BAC) clone, RP11-1122L19 (chr15:55,589,601-55,722,445, GRCh38/Hg38), was obtained from BACPAC Resources Center (Oakland, CA, USA). BAC DNA was extracted using QIAGEN Plasmid Midi Kit (QIAGEN, Germantown, MD, USA) and labeled with SpetrumGreen using Abbott Nick Translation Labeling Kit (Abbott Molecular, Des Plaines, IL, USA) both following the kit manuals. FISH results were analyzed and documented using the CytoVision system (Leica Microsystems, San Jose, CA, USA).

### 2.3. Whole-Genome MP-Seq

Genomic DNA was extracted from bone marrow aspirate using the QIAGEN Amp mini blood DNA kit (QIAGEN, Germantown, MD, USA). MP-seq library was prepared using Nextera Mate Pair Sample Preparation Kit following the kit manual (Illumina, San Diego, CA, USA). The library was pair-end sequenced (PE150, 150 × 2 bp, ~10× genome coverage) at Novogene (Sacramento, CA, USA). DNA sequencing data were analyzed for structural and copy number changes by WholeGenome, LLC (Rochester, MN, USA).

### 2.4. Polymerase Chain Reaction (PCR) and Sanger Sequencing

To confirm the MP-seq findings and to identify the exact DNA breakpoint sequences, six pairs of PCR primers were designed using Prime3 (https://primer3.ut.ee/, accessed on 19 May 2021) and synthesized at Millipore Sigma (St. Louis, MO, USA):

1. TGGGGAGCCCTTATTATTTTT and TTCTATCCCCAAGCCTTCCT for der(1) of t(1;15)(p34;q15),
2. CCACATAATCAAAAGTTGACTGC and ACGGCATGAGTCCAGAAGAT for der(15) of t(1;15)(p34;q15),
3. ACGCAGCCTTCACTGGTAGT and CTGTCTGCATGTAAACTGTAT for del(15q),
4. TTTGTTTTTAGGCAGGCAAA and CAAGACTTTCTGCCCCAATG for der(9) of t(9;12)(q34;p13),
5. CAGAGGCAGATAAAAATTCTCCA and GGAAAAGTTTGCCGGATACA for der(12) of t(9;12)(q34;p13),
6. CTGGGATCCCCATCCTATT and AGATGAGCAACCCAAGCATC for the proximal breakpoint of inv(12p).

PCRs were performed using a standard PCR protocol. PCR products were purified and sequenced at MCLAB (South San Francisco, CA, USA).

## 3. Case Presentation

A 51-year-old male presented with progressive fatigue, weight loss, and splenomegaly. Peripheral blood analysis revealed leukocytosis, thrombocytosis, monocytosis, and anemia. Peripheral blood counts showed white blood cells 241 × 10^9^/L, absolute eosinophils 2.41 × 10^9^/L, hemoglobin 6 g/dL, and platelets 50 × 10^9^/L. An increased proportion of both eosinophilic and basophilic precursors was noted in the bone marrow aspirate smears. His bone marrow showed morphological features compatible with both myelodysplastic syndrome and myeloproliferative neoplasm (MDS/MPN). Among the MDS/MPN overlap neoplasms, chronic myelomonocytic leukemia, atypical chronic myeloid leukemia, and unclassifiable MDS/MPN were considered. The patient tested positive for *ETV6*::*ABL1* gene fusion, suggesting that he could benefit from TKI therapy. He was treated with imatinib for three months, and then switched to dasatinib due to the persistence of myeloid neoplasm. He reached complete morphological remission after dasatinib therapy, and subsequently underwent allogeneic stem cell transplantation from a matched unrelated donor. The patient has been in complete remission for more than 13 years following the transplantation.

## 4. Results

### 4.1. Initial Genomic Testing

Multiple genetic tests, including chromosome analysis, FISH for *BCR*::*ABL1* gene fusion, array comparative genomic hybridization (aCGH) and *JAK2* mutation analysis, were performed based on the patient’s clinical and pathological findings. A balanced translocation between chromosomes 1 and 15 and an apparent rearrangement involving a distal region on the short arm of a chromosome 12 (12p) were detected in all 20 metaphase cells analyzed. Overall, 3 of these 20 cells also showed an insertion of a fragment from the long arm of a chromosome 8 into the long arm of a chromosome 15 that was not involved in the t(1;15) translocation, suggesting cytogenetic evolution. Interphase FISH analysis did not detect a typical *BCR*::*ABL1* gene fusion signal pattern, but instead the FISH showed an additional *ABL1* signal in approximately 41.5% of the cells examined. The nomenclature of the initial chromosome and FISH testing results was: 46,XY,t(1;15)(p34.2;q15),?add(12)(p13)[17]/46,idem,ins(15;8)(q14;q13q22)[3].nuc ish(*ABL1*x3,BCRx2)[83/200]. In addition, aCGH (sent-out test) revealed a 522.4kb interstitial deletion within the band 15q21.3 on the long arm of chromosome 15, involving the *PYGO1*, *PRTG*, *NEDD4*, *RFXDC2*, and *RFX7* genes. JAK2 V617F mutation (sent-out test) was negative.

### 4.2. Verifications

**Figure 1:**
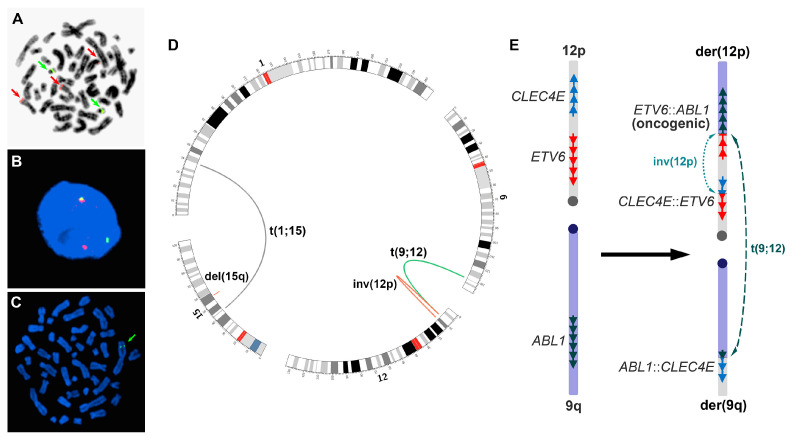
(**A**) Metaphase FISH using a *BCR*/*ABL1* Dual Color Dual Fusion probe set, showing the extra *ABL1* signal on 12p (top red arrow); red arrow—*ABL1* signal, green arrow—*BCR* signal. (**B**) Interphase FISH using an *ETV6* Dual Color Break Apart probe set, showing *ETV6* rearrangement; red signal—5′*ETV6*, green signal—3′*ETV6*. (**C**) Metaphase FISH using probe RP11-1122L19 within the 15q21.3 region (green arrow), showing that the region was present only on the 15q that was involved in the t(1;15) translocation. (**D**) The genomic rearrangements detected by MP-seq (the figure was drawn using Circos, http://circos.ca, accessed on 28 May 2022). (**E**) Left: gene orientations on chromosomes 12p and 9q before the rearrangements; right: the paracentric inversion of 12p, translocation between 9q and the inverted 12p, and three in-frame fusion genes resulted from the inversion and translocation. The inversion and the translocation could occur simultaneously or sequentially.

Metaphase FISH was performed to verify the interphase *BCR*::*ABL1* FISH findings. It showed that the extra *ABL1* signal was located on the short arm of a chromosome 12 in the proximity of the *ETV6* gene locus, suggesting the possibility of *ETV6*::*ABL1* gene fusion (Figure 1A). The fusion was confirmed by additional genomic tests, including MP-seq (Figure 1D and Supplementary Figure S1A) and interphase FISH that showed *ETV6* gene rearrangement (Figure 1B). The MP-seq also demonstrated that the in-frame *ETV6*::*ABL1* fusion was formed through two rearrangements, including a 3.342 Mb paracentric inversion on 12p between the *CLEC4E* and *ETV6* genes (chr12: 8,539,067-11,881,857, GRCh38/hg38) and a translocation between the inverted *ETV6* on 12p and *ABL1* on 9q (Figure 1D,E). The in-frame *ETV6*::*ABL1* fusion gene contained *ETV6* exons 1-5 and *ABL1* exons 2-11. However, whether the two rearrangements occurred consecutively or simultaneously was unclear.

The MP-seq also confirmed the t(1;15) translocation and the 15q interstitial deletion (chr15:55,553,970-56,099,049, GRCh38/hg38) (Figure 1D and Figure S1A), and revealed additional in-frame fusion genes and interrupted genes due to the chromosomal rearrangements. The in-frame fusions included *CLEC4E*::*ETV6* (*CLEC4E* exons 1-3 and *ETV6* exons 6-8, resulting from the inversion of 12p), *ABL1*::*CLEC4E* (*ABL1* exon 1 and *CLEC4E* exons 4-6, resulting from the translocation between 9q and 12p) and *RFX7*::*PYGO1* (*RFX7* exons 1-7 and *PYGO1* exons 2-3, resulting from the 15q deletion). The interrupted genes included *HEYL* and *ZNF106* due to the t(1;15) translocation. All breakpoints that the MP-seq detected were confirmed by PCR and Sanger sequencing, and various deletions ranging from 15~992 bp in size were also detected at the breakpoints (Figure S1). Metaphase FISH further demonstrated that the 15q deletion occurred on the chromosome 15 homologue that was not involved in the t(1;15) translocation (Figure 1C).

### 4.3. Following-Up Studies

Following initiation of TKI therapy, chromosome analysis and interphase FISH for *ABL1* rearrangement were regularly performed to monitor the patient’s response. The t(1;15) translocation and *ABL1* rearrangement were detected in the patient’s marrow after three month of imatinib therapy along with persistent morphologic evidence of myeloid neoplasm. The patient was then switched to dasatinib, and after three months achieved a morphological marrow remission. No evidence of the *ABL1* rearrangement was detected by FISH in two consecutive following-up studies after the initiation of dasatinib therapy. However, the t(1;15) translocation was detected in approximately 7.0% and 24.0% of the metaphase cells in the first and second following-up tests after the dasatinib therapy, respectively. The remaining cells had a normal male karyotype. The patient then underwent allogeneic stem cell transplantation from a matched unrelated male donor due to a concern that the cells with persistent t(1;15) could post a risk of relapse. He has remained disease-free for more than 13 years following the transplantation.

## 5. Discussion

The formation of an in-frame *ETV6*::*ABL1* fusion gene involves complex genomic rearrangements due to opposite chromosome orientations of the *ETV6* and *ABL1* genes. However, only limited studies of the fusion mechanisms at the chromosome level have been reported. Information regarding the genomic rearrangement, content, and breakpoints involving in the fusion at the molecular level is lacking. To our knowledge, four mechanisms regarding the formation of in-frame *ETV6*::*ABL1* gene fusion have been reported, including the insertion of 5′*ETV6* into *ABL1* [11,12,13,14], the insertion of 3′*ABL1* into *ETV6* [15,16,17,18], the inversion of 9q (reversing *ABL1*) in combination with a t(9;12) translocation [19,20], and *ETV6* insertion and translocation involving multiple chromosomes [21]. Except for chromosomal findings, however, all these mechanisms lacked molecular evidence at the DNA level. In the present case, we identified another mechanism at the DNA level through detailed genomic analysis of the neoplastic cells. The in-frame *ETV6*::*ABL1* fusion in this case was formed via a paracentric inversion on chromosome 12p and a translocation between chromosome 9q and the inverted 12p (Figure 1E). The inversion reverses the orientation of *ETV6* exons 1-5; the precise breakpoints, nucleotide joining points and gene contents involved in the rearrangements were clearly defined (Figure S1). However, the data from our studies were not sufficient to determine whether the inversion and translocation occurred sequentially or simultaneously. A similar mechanism was only speculated but not verified with direct molecular evidence [22,23].

We detected multiple genomic anomalies in the patient’s initial bone marrow specimen. Two of them, t(1;15) translocation and *ABL1* rearrangement that represented the *ETV6*::*ABL1* gene fusion, were regularly tested in the following-up studies to monitor the patient’s therapy and prognosis. After initiation of dasatinib therapy, the *ABL1* rearrangement was undetectable in two consecutive following-up FISH tests, consistent with the morphological remission of the patient. However, the t(1;15) translocation was still present in 7.0% and 24.0% of the cells in the first and second follow-up tests, respectively, which is inconsistent with the morphological findings. These results indicated two abnormal cell populations: one with both the translocation and *ABL1* rearrangement, and the other with the translocation only. We speculate that the t(1;15) translocation occurred prior to the *ETV6*::*ABL1* fusion, followed by the development of the fusion in a subset of the cells with the translocation, thus leading to neoplastic transformation. At diagnosis, the neoplastic clone with t(1;15) and *ETV6*::*ABL1* fusion was likely the predominant cell population that overgrew the cells without the fusion in the patient’s bone marrow before therapy. After the effective therapy, the neoplastic clone with the *ETV6*::*ABL1* fusion was eliminated, and the cells without the fusion, including the cells with and without the t(1;15) translocation, survived and grew back.

It is noteworthy that multiple genes were affected by the t(1:15) translocation and the co-occurred interstitial deletion of 15q (see Section 4.1 and Section 4.2), and some of these genes are apparently related to tumorigenesis or tissue development pathways. The *RFX7* gene encodes a transcription factor regulated by TP53 and targeting multiple known tumor suppressors. Mutations in *RFX7* have recurrently been reported in lymphoid cancers [24,25,26]. The *PYGO1* gene encodes a nuclear protein essential for Wnt signal pathway, and deregulation of Wnt pathway is a well-known cause of cancer [27,28,29]. NEDD4, an E3 ubiquitin ligase, has been suggested to play important roles in cancer as the tumor suppressor protein PTEN is a direct target of NEDD4 ubiquination [30,31]. In addition, HEYL is a helix–loop–helix transcription factor, and PRTG has been associated with the development of various tissues [32,33]. Based on these findings, we hypothesize that the t(1;15) translocation and/or the deletion of 15q might represent a predisposing genomic lesion, although the pathogenic roles of the translocation and the deletion need to be verified. As expected, dasatinib therapy was only effective at treating the neoplastic cells with the *ETV6*::*ABL1* fusion, but not the cells without the fusion. To our knowledge, the development of an oncogenic *ETV6*::*ABL1* fusion in a subset of predisposed cells has not been reported. In addition, as there are no standard clinical tests to detect *ETV6*::*ABL1* fusions, this case highlights the potential therapeutic importance of investigating abnormal *ABL1* fusions that do not involve *BCR*.

Additional anomalies co-occurring with *ETV6*::*ABL1* fusion are frequent, but their significance is yet to be understood. The multiple and complex anomalies seen in this case suggested genomic instability that might play a critical role in the genesis of the *ETV6*::*ABL1* gene fusion. We speculate that some additional anomalies could predispose cells to additional pathogenic genomic alterations and neoplastic transformation. In this case, it is unclear whether the additional anomalies played a role in the patient’s clinical presentation, therapy response, and prognosis. The t(1;15) translocation and deletion of 15q could represent somatic or mosaic constitutional changes, as constitutional analysis was not performed.

## 6. Conclusions

In summary, we characterized genomic anomalies in an individual with *ETV6*::*ABL1* gene-fusion-positive myeloid neoplasm using various genomic technologies. The oncogenic in-frame *ETV6*::*ABL1* gene fusion was identified following the detection of *ABL1* and *ETV6* rearrangements via FISH studies. The identification of the fusion played a critical role in the decision to employ ABL1 TKI therapy, which achieved morphologic remission, thereby facilitating a successful allogeneic stem cell transplantation procedure. The *ETV6*::*ABL1* gene fusion was formed through a paracentric inversion of 12p and a translocation between 9q and the 12p with the inversion. This *ETV6*::*ABL1* fusion apparently occurred as a secondary event in a subset of cells that may have been predisposed to develop a complex genomic abnormality due to a pre-existing lesion.

## Data Availability

The data that support the findings of this study are available from the corresponding author upon request.

## Supplementary Materials

The following supporting information can be downloaded at: https://www.mdpi.com/article/10.3390/genes14101851/s1, Figure S1: DNA breakpoints determined by MP-seq and Sanger sequencing. (A) MP-seq reads (line-connected dots), indicating joining breakpoints from different chromosomes or chromosome regions. (B) Confirmation of the MP-seq findings by Sanger sequencing of PCR products that were amplified crossing the joining breakpoints of the rearrangements. The black vertical lines indicate the breakpoints. (C) DNA breakpoint locations on the chromosomes involved. Deletions (dotted line) were found at each breakpoint. Black line: intron; black box: exon.

## References

[1] Andreasson P., Johansson B., Carlsson M., Jarlsfelt I., Fioretos T., Mitelman F., Hoglund M. (1997). BCR/ABL-negative chronic myeloid leukemia with ETV6/ABL fusion. Genes Chromosomes Cancer.

[2] La Starza R., Trubia M., Testoni N., Ottaviani E., Belloni E., Crescenzi B., Martelli M., Flandrin G., Pelicci P.G., Mecucci C. (2002). Clonal eosinophils are a morphologic hallmark of ETV6/ABL1 positive acute myeloid leukemia. Haematologica.

[3] Uemura S., Nishimura N., Hasegawa D., Shono A., Sakaguchi K., Matsumoto H., Nakamachi Y., Saegusa J., Yokoi T., Tahara T. (2018). ETV6-ABL1 fusion combined with monosomy 7 in childhood B-precursor acute lymphoblastic leukemia. Int. J. Hematol..

[4] Yamamoto K., Yakushijin K., Nakamachi Y., Miyata Y., Sanada Y., Tanaka Y., Okamura A., Kawano S., Hayashi Y., Matsuoka H. (2014). Extramedullary T-lymphoid blast crisis of an ETV6/ABL1-positive myeloproliferative neoplasm with t(9;12)(q34;p13) and t(7;14)(p13;q11.2). Ann. Hematol..

[5] Zaliova M., Moorman A.V., Cazzaniga G., Stanulla M., Harvey R.C., Roberts K.G., Heatley S.L., Loh M.L., Konopleva M., Chen I.M. (2016). Characterization of leukemias with ETV6-ABL1 fusion. Haematologica.

[6] Yao J., Xu L., Aypar U., Meyerson H.J., Londono D., Gao Q., Baik J., Dietz J., Benayed R., Sigler A. (2021). Myeloid/lymphoid neoplasms with eosinophilia/ basophilia and ETV6-ABL1 fusion: Cell-of-origin and response to tyrosine kinase inhibition. Haematologica.

[7] Schwaab J., Naumann N., Luebke J., Jawhar M., Somervaille T.C.P., Williams M.S., Frewin R., Jost P.J., Lichtenegger F.S., La Rosee P. (2020). Response to tyrosine kinase inhibitors in myeloid neoplasms associated with PCM1-JAK2, BCR-JAK2 and ETV6-ABL1 fusion genes. Am. J. Hematol..

[8] Perna F., Abdel-Wahab O., Levine R.L., Jhanwar S.C., Imada K., Nimer S.D. (2011). ETV6-ABL1-positive “chronic myeloid leukemia”: Clinical and molecular response to tyrosine kinase inhibition. Haematologica.

[9] Lin H., Guo J.Q., Andreeff M., Arlinghaus R.B. (2002). Detection of dual TEL-ABL transcripts and a Tel-Abl protein containing phosphotyrosine in a chronic myeloid leukemia patient. Leukemia.

[10] Jordan M., Hastings R.J., Moore S. (2020). ISCN 2020: An International System for Human Cytogenomic Nomenclature.

[11] Van Limbergen H., Beverloo H.B., van Drunen E., Janssens A., Hahlen K., Poppe B., Van Roy N., Marynen P., De Paepe A., Slater R. (2001). Molecular cytogenetic and clinical findings in ETV6/ABL1-positive leukemia. Genes Chromosomes Cancer.

[12] Kakadia P.M., Schmidmaier R., Volkl A., Schneider I., Huk N., Schneider S., Panzner G., Neidel U., Fritz B., Spiekermann K. (2016). An ETV6-ABL1 fusion in a patient with chronic myeloproliferative neoplasm: Initial response to Imatinib followed by rapid transformation into ALL. Leuk. Res. Rep..

[13] Choi S.I., Jang M.A., Jeong W.J., Jeon B.R., Lee Y.W., Shin H.B., Hong D.S., Lee Y.K. (2017). A Case of Chronic Myeloid Leukemia With Rare Variant ETV6/ABL1 Rearrangement. Ann. Lab. Med..

[14] Lukes J., Potuckova E., Sramkova L., Stary J., Starkova J., Trka J., Votava F., Zuna J., Zaliova M. (2018). Two novel fusion genes, AIF1L-ETV6 and ABL1-AIF1L, result together with ETV6-ABL1 from a single chromosomal rearrangement in acute lymphoblastic leukemia with prenatal origin. Genes Chromosomes Cancer.

[15] Renzi S., Algawahmed F., Davidson S., Langenberg K.P.S., Fuligni F., Ali S., Anderson N., Brunga L., Bartram J., Abdelhaleem M. (2023). Myeloproliferative Neoplasm Driven by ETV6-ABL1 in an Adolescent with Recent History of Burkitt Leukemia. Curr. Oncol..

[16] Park J., Kim M., Lim J., Kim Y., Han K., Kim J.S., Lee S., Kim H.J., Min W.S. (2013). Variant of ETV6/ABL1 gene is associated with leukemia phenotype. Acta Haematol..

[17] Kelly J.C., Shahbazi N., Scheerle J., Jahn J., Suchen S., Christacos N.C., Mowrey P.N., Witt M.H., Hostetter A., Meloni-Ehrig A.M. (2009). Insertion (12;9)(p13;q34q34): A cryptic rearrangement involving ABL1/ETV6 fusion in a patient with Philadelphia-negative chronic myeloid leukemia. Cancer Genet. Cytogenet..

[18] Baeumler J., Szuhai K., Falkenburg J.H., van Schie M.L., Ottmann O.G., Nijmeijer B.A. (2008). Establishment and cytogenetic characterization of a human acute lymphoblastic leukemia cell line (ALL-VG) with ETV6/ABL1 rearrangement. Cancer Genet. Cytogenet..

[19] Gancheva K., Virchis A., Howard-Reeves J., Cross N.C., Brazma D., Grace C., Kotzampaltiris P., Partheniou F., Nacheva E. (2013). Myeloproliferative neoplasm with ETV6-ABL1 fusion: A case report and literature review. Mol. Cytogenet..

[20] Barbouti A., Ahlgren T., Johansson B., Hoglund M., Lassen C., Turesson I., Mitelman F., Fioretos T. (2003). Clinical and genetic studies of ETV6/ABL1-positive chronic myeloid leukaemia in blast crisis treated with imatinib mesylate. Br. J. Haematol..

[21] Tirado C.A., Siangchin K., Shabsovich D.S., Sharifian M., Schiller G. (2016). A novel three-way rearrangement involving ETV6 (12p13) and ABL1 (9q34) with an unknown partner on 3p25 resulting in a possible ETV6-ABL1 fusion in a patient with acute myeloid leukemia: A case report and a review of the literature. Biomark. Res..

[22] Song J.S., Shin S.Y., Lee S.T., Kim H.J., Kim S.H. (2014). A cryptic ETV6/ABL1 rearrangement represents a unique fluorescence in situ hybridization signal pattern in a patient with B acute lymphoblastic leukemia. Ann. Lab. Med..

[23] Mori N., Ohwashi-Miyazaki M., Okada M., Yoshinaga K., Shiseki M., Tanaka J. (2016). Translocation (9;12)(q34.1;p13.?3) Resulted in ETV6-ABL1 Fusion in a Patient with Philadelphia Chromosome-Negative Chronic Myelogenous Leukemia. Acta Haematol..

[24] Fischer B.A., Chelbi S.T., Guarda G. (2020). Regulatory Factor X 7 and its Potential Link to Lymphoid Cancers. Trends Cancer.

[25] Weber J., de la Rosa J., Grove C.S., Schick M., Rad L., Baranov O., Strong A., Pfaus A., Friedrich M.J., Engleitner T. (2019). PiggyBac transposon tools for recessive screening identify B-cell lymphoma drivers in mice. Nat. Commun..

[26] Coronel L., Riege K., Schwab K., Forste S., Hackes D., Semerau L., Bernhart S.H., Siebert R., Hoffmann S., Fischer M. (2021). Transcription factor RFX7 governs a tumor suppressor network in response to p53 and stress. Nucleic Acids Res..

[27] Jessen S., Gu B., Dai X. (2008). Pygopus and the Wnt signaling pathway: A diverse set of connections. BioEssays News Rev. Mol. Cell. Dev. Biol..

[28] Kramps T., Peter O., Brunner E., Nellen D., Froesch B., Chatterjee S., Murone M., Zullig S., Basler K. (2002). Wnt/wingless signaling requires BCL9/legless-mediated recruitment of pygopus to the nuclear β-catenin-TCF complex. Cell.

[29] Belenkaya T.Y., Han C., Standley H.J., Lin X., Houston D.W., Heasman J., Lin X. (2002). pygopus Encodes a nuclear protein essential for wingless/Wnt signaling. Development.

[30] Boase N.A., Kumar S. (2015). NEDD4: The founding member of a family of ubiquitin-protein ligases. Gene.

[31] Chen C., Matesic L.E. (2007). The Nedd4-like family of E3 ubiquitin ligases and cancer. Cancer Metastasis Rev..

[32] Steidl C., Leimeister C., Klamt B., Maier M., Nanda I., Dixon M., Clarke R., Schmid M., Gessler M. (2000). Characterization of the human and mouse HEY1, HEY2, and HEYL genes: Cloning, mapping, and mutation screening of a new bHLH gene family. Genomics.

[33] Toyoda R., Nakamura H., Watanabe Y. (2005). Identification of protogenin, a novel immunoglobulin superfamily gene expressed during early chick embryogenesis. Gene Expr. Patterns.

