# Supplementary material for: Complex Genomic Rearrangements Involving ETV6::ABL1 Gene Fusion in an Individual with Myeloid Neoplasm
Source: Genes (Basel). 2023 Sep 23;14(10):1851. doi: 10.3390/genes14101851 (PMC10606058; doi:10.3390/genes14101851)

Supplementary Figure 1. DNA breakpoints determined by MP-seq and Sanger sequencing. (A) MP-seq reads (line-connected dots), indicating joining breakpoints from different chromosomes or chromosome regions. (B) Confirmation of the MP-seq findings by Sanger sequencing of PCR products that were amplified crossing the joining breakpoints of the rearrangements. The black vertical lines indicate the breakpoints. (C) DNA breakpoint locations on the chromosomes involved. Deletions (dotted line) were found at each breakpoint. Black line: intron; black box: exon.

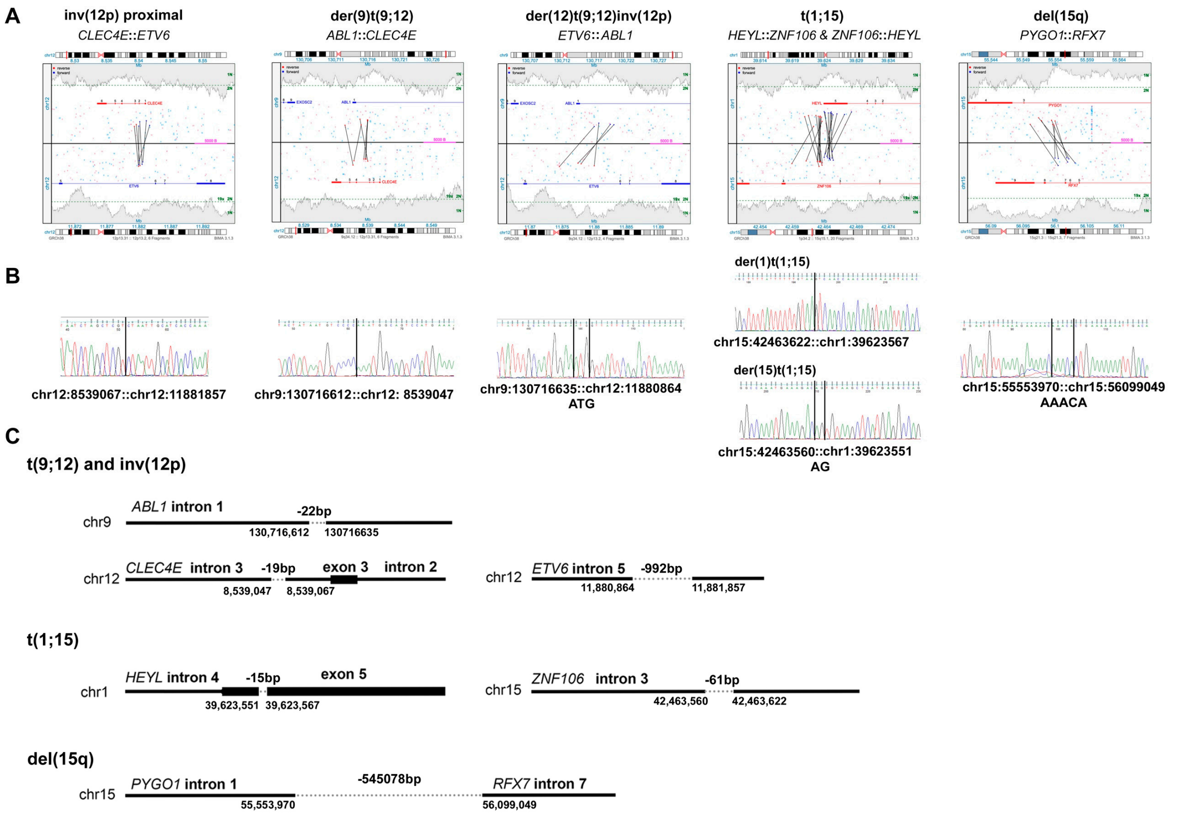

Supplement: Supplementary file 1 [file genes-14-01851-s001.zip › genes-2581610-supplementary.pdf]
